# Parkinsonian phenotypes induced by Synphilin-1 expression are differentially contributed by serotonergic and dopaminergic circuits and suppressed by nicotine treatment

**DOI:** 10.1371/journal.pone.0282348

**Published:** 2023-03-01

**Authors:** Angel Carvajal-Oliveros, Carmen Dominguez-Baleón, Iván Sánchez-Díaz, Diego Zambrano-Tipan, René Hernández-Vargas, Jorge M. Campusano, Verónica Narváez-Padilla, Enrique Reynaud

**Affiliations:** 1 Departamento de Genética del Desarrollo y Fisiología Molecular, Instituto de Biotecnología, Universidad Nacional Autónoma de México, Cuernavaca, México; 2 Facultad de Ciencias Biológicas, Departamento de Biología Celular y Molecular, Laboratorio Neurogenética de la Conducta, Pontificia Universidad Católica de Chile, Santiago, Chile; 3 Centro de Investigación en Dinámica Celular, Universidad Autónoma del Estado de Morelos, Cuernavaca, Morelos, México; Biomedical Sciences Research Center Alexander Fleming, GREECE

## Abstract

Synphilin-1 is a protein encoded by the human SNCAIP gene whose function has yet to be fully understood. However, it has been linked to familial Parkinson’s disease (PD). Synphilin-1 is a major component of the Lewy bodies found in neurons in the *substantia nigra pars compacta* of PD patients. Synphilin-1 expression in serotonergic and/or dopaminergic neurons of *Drosophila melanogaster* induces neurodegeneration, as well as motor and non-motor PD like symptoms. In this work, we examined the contribution of the serotonergic and dopaminergic circuits in the development of PD-like phenotypes. We found that olfactory and visual symptoms are majorly contributed by the serotonergic system, and that motor symptoms and reduction in survival are mainly contributed by the dopaminergic system. Chronic nicotine treatment was able to suppress several of these symptoms. These results indicate that both the serotonergic and dopaminergic systems contribute to different aspects of PD symptomatology and that nicotine has beneficial effects on specific symptoms.

## Introduction

Parkinson’s disease (PD) is a neurodegenerative disorder characterized by the loss of dopaminergic neurons in the *substantia nigra pars compacta*, leading to a reduction in dopamine availability in the *striatum* and resulting in a range of symptoms including postural instability, mobility loss, bradykinesia, olfactory abnormalities, and sleep disorders [1,2]. Non-motor symptoms, including olfactory dysfunction, vision changes, depression, and sleep disorders, often occur years or even decades before the onset of motor symptoms [3–5]. These symptoms have been linked to dysregulation of biogenic amine circuits, particularly the dopaminergic system, but other aminergic neural systems, such as the serotonergic system, may also be involved [6–9].

Synphilin-1 (Sph-1) is a cytoplasmic protein encoded by the human SNCAIP gene. While the precise functions of Sph-1 are not yet fully understood, it is known to be highly enriched in presynaptic terminals [10]. Sph-1 is also a major component of Lewy bodies, intracellular protein inclusions that have been directly associated with the development and progression of neurodegenerative diseases [11]. Sph-1 has at least eight isoforms, with the shorter ones more prone to aggregation and thought to be involved in neuropathic processes [12,13].

We have previously shown that the expression of Sph-1 or α-synuclein in *Drosophila melanogaster* leads to premature mortality and extensive neurodegeneration, as well as PD-like motor phenotypes [14,15]. Interestingly, we also found early olfactory abnormalities, a common early symptom of PD [16]. In our previous work we also found that the expression of Sph-1 in neurons caused stronger phenotypes than α-synuclein expression, but co-expression of these two proteins mutually suppressed their toxicity. Additionally, expression of Sph-1 in fly dopaminergic neurons reduced total brain tyrosine hydroxylase (TH) protein levels and dopamine content, but nicotine, a cholinergic agonist and natural insecticide found in tobacco, reversed Sph-1 phenotypes and increased dopamine production in Sph-1-expressing brains [14,15].

In this study, we further characterized the parkinsonian phenotypes associated with the expression of Sph-1 in dopaminergic and serotonergic neurons of the *Drosophila* brain, and explored whether dopaminergic and serotonergic circuits differentially contribute to the parkinsonian phenotypes induced by Sph-1 expression. Our results show that nicotine increased the lifespan of flies regardless of whether Sph-1 was expressed in dopaminergic or serotonergic neurons. Our data is consistent with previous literature that shows that serotonin is involved in olfactory modulation [16,17] indicating that while Sph-1-induced alterations in olfactory response depend mostly on the serotonergic system, motor phenotypes are largely explained by Sph-1 expression in the dopaminergic system. Olfactory and motor alterations were suppressed by chronic nicotine treatment. Finally, we found that Sph-1 expression in either aminergic system affected electroretinographic parameters, which were also rescued by chronic nicotine treatment.

## Results

### Sph-1 expression in the serotonergic system does not affect life span while its expression in the dopaminergic system significantly reduces survival

**Fig 1:**
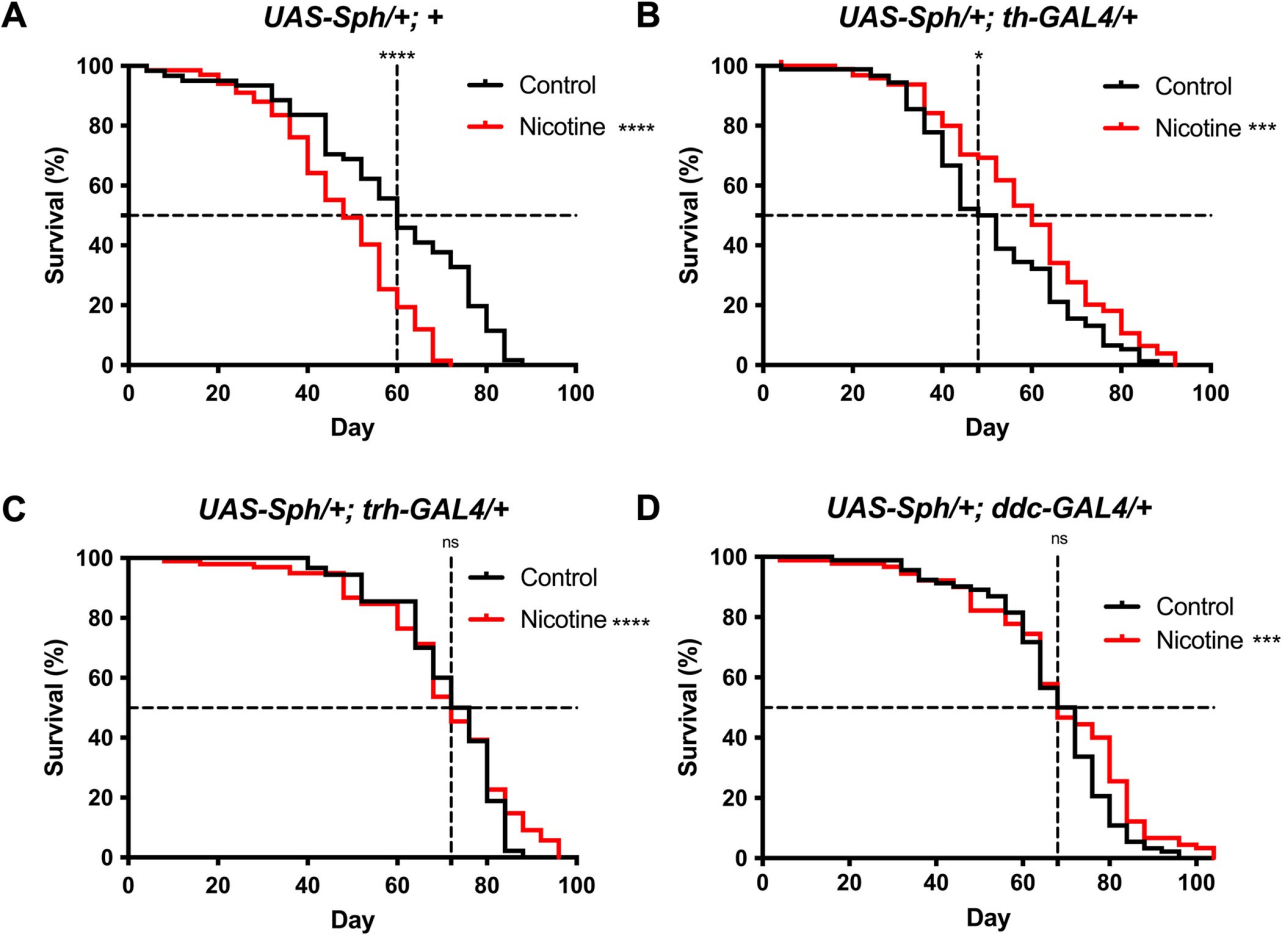
The dopaminergic system contributes to fly survival and is the neurochemical system responsible for the nicotine protective effects. A) Nicotine-treated (24 μM) control flies that do not express Sph-1 (UAS-Sph-1/+; +) show reduced life expectancy compared to non-treated animals. B) Flies that express Sph-1 in dopaminergic neurons (UAS-Sph-1/+; +) have a reduced life expectancy that is rescued by nicotine treatment. C) In flies that express Sph-1 in their serotonergic system (*UAS-Sph-1/+; UAS-tph-GAL4/+*), the effect of nicotine on life span is smaller; however, this chemical significantly extends life expectancy, particularly at older ages (around 80 days). D) When Sph-1 is expressed in both the dopaminergic and serotonergic systems (*UAS-Sph-1/+; UAS-ddc-GAL4/+*), nicotine treatment promotes survival in a similar manner to that observed in flies expressing Sph-1 only in the dopaminergic system. The most evident effect is in flies aged over 60 days. Whole lifespan significance was evaluated using the Mantel-Cox test. Asterisks denote significance between untreated (black lines) and treated (red lines) groups, *** P < 0.001, **** P < 0.0001; n = 100 animals per group. Significant differences at half-lives (asterisks over the vertical dotted line) were assessed using Fisher’s exact test. Control P = 0.0142, TH P = 0.0374, TPH P = 0.3091, DDC P = 0.882.

Previous research has shown that the expression of Sph-1 in dopaminergic neurons significantly reduces *Drosophila* survival, and this effect can be suppressed by chronic nicotine treatment [15]. Therefore, we sought to investigate whether the expression of Sph-1 in serotonergic neurons would have any effect on fly lifespan. We used three different genotypes to express Sph-1 in serotonergic and/or dopaminergic neurons. The genotype *UAS-Sph1/+; tph-GAL4/+* used the tryptophan hydroxylase (*tph-GAL4*) GAL4 driver to express Sph-1 in serotonergic neurons, while the genotype U*AS-Sph1/+; ddc-GAL4/+* used the Dopa-decarboxylase GAL4 driver (*ddc-GAL4*) to express Sph-1 in both dopaminergic and serotonergic neurons. This GAL4 driver has an enhancer of the Ddc gene, which drives expression in both types of aminergic neurons [18]. The genotype *UAS-Sph1/+; th-GAL4* used the tyrosine hydroxylase driver (*th-GAL4*) to express Sph-1 only in dopaminergic neurons. In control flies that did not express Sph-1 (*UAS-Sph1/+*), chronic nicotine treatment significantly reduced both half-life and maximum lifespan (**Fig 1A**). Flies with a parkinsonian genotype that expressed Sph-1 in dopaminergic neurons (*UAS-Sph1/+; th-GAL4*) showed a reduction in lifespan that was rescued by chronic nicotine treatment, with the largest effect observed at 60 days post-eclosion (**Fig 1B**). In contrast, the expression of Sph-1 in serotonergic neurons (*UAS-Sph1/+; tph-GAL4*) had no significant effect on lifespan, although nicotine treatment did lead to a modest but significant increase in maximum life expectancy. When Sph-1 expression was driven in both dopaminergic and serotonergic neurons using the Dopa-decarboxylase (DDC) driver (*UAS-Sph1/+; ddc-GAL4*), the effect of nicotine treatment on fly survival was smaller and observed later in the life of the animals, most evident at 80 days post-eclosion (**Fig 1C and 1D**). However, the effect was still statistically significant, suggesting that Sph-1 is more toxic or disruptive in the dopaminergic system and that there is some interaction between the two neural circuits that modulates fly survival (**Fig 1C and 1D**).

### The serotonergic system is responsible for most of the Sph-1 induced olfactory alterations observed in young flies

**Fig 2:**
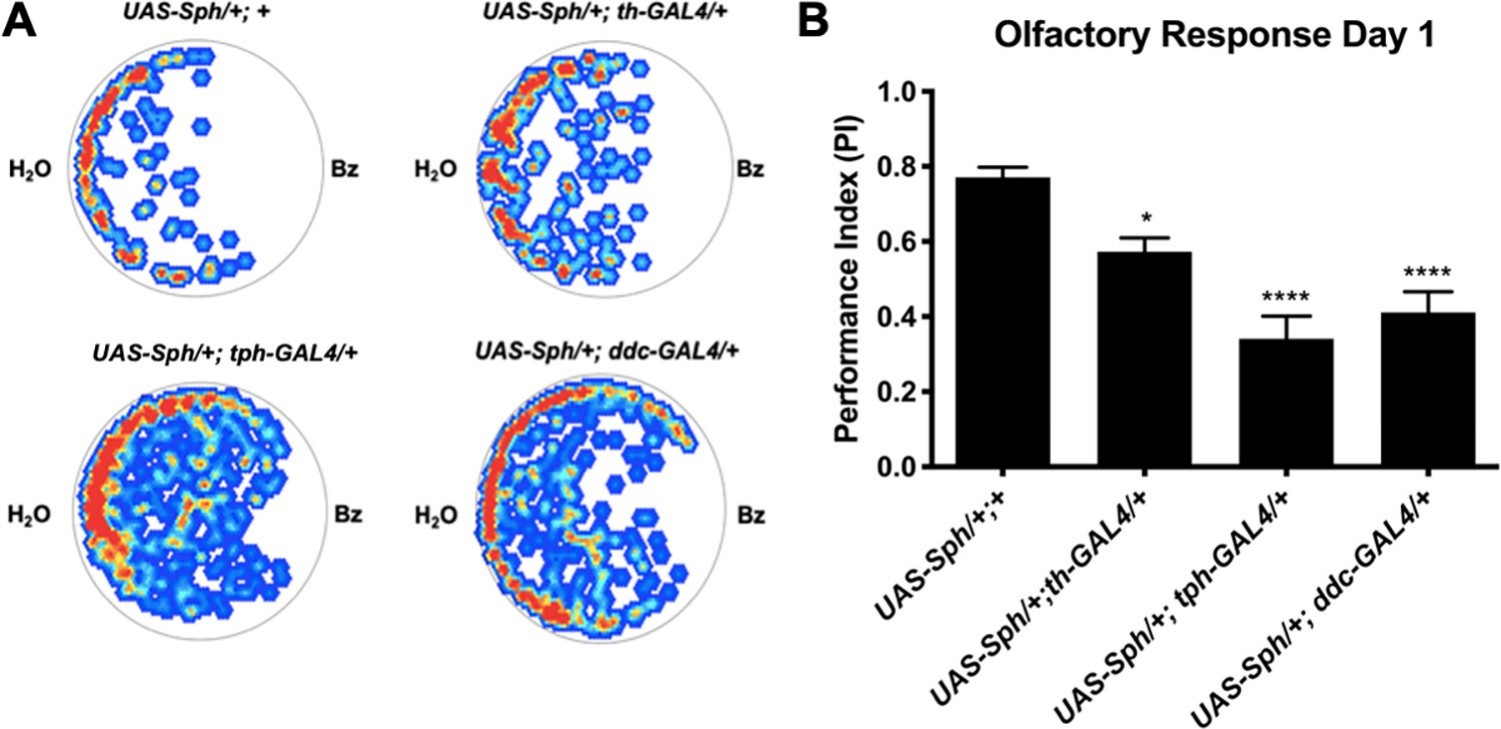
Young flies that express Sph-1 in their dopaminergic and/or serotonergic systems exhibit reduced olfactory response. A) Representative heat maps showing the response of day 1 post-eclosion flies to the repulsive odorant benzaldehyde (Bz). Red dots indicate areas where the fly spent more time, while blue dots indicate areas where they spent less time; white areas were mostly avoided. B) Quantification of the observed response to Bz in day 1 post-eclosion flies. Data show that flies that express Sph-1 in either the dopaminergic or serotonergic neural systems, or in both, exhibit an attenuated aversive response compared to controls that do not express Sph-1. However, the effect of Sph-1 appears to be greater in flies expressing Sph-1 in the serotonergic system. Significance was tested using two-way ANOVAs followed by a Tukey post-hoc test, * P < 0.05, ** P < 0.01, *** P < 0.001, **** P < 0.0001, ns = not significant; n = 20 animals per condition.

Non-motor symptoms such as olfactory impairments are very relevant because they could be used as early indicators of PD. In a previous study, we found that the expression of Sph-1 in dopaminergic neurons significantly decreased the olfactory response to an aversive odor in young flies [15]. The serotonergic system has been shown to play a role in olfactory processing in both mammals and *Drosophila* [16]. Therefore, we decided to investigate the contribution of the serotonergic system to olfactory impairment in PD. We found that the simultaneous expression of Sph-1 in both dopaminergic and serotonergic neurons, or solely in serotonergic cells, resulted in a stronger olfactory defect than when Sph-1 was only expressed in dopaminergic neurons (**Fig 2A and 2B**). These results suggest that alterations in the serotonergic system have a greater impact on olfactory dysfunction in PD than the dopaminergic system.

### The dopaminergic system is mainly involved in the motor symptoms induced by Sph-1 expression

Motor symptoms are the most notable features of PD. It has been suggested that these motor abnormalities depend not only on dopaminergic neurons, but also on other neurochemical systems [9,19]. We aimed to investigate the effect of Sph-1 expression on motor behavior in a PD fly model.

**Fig 3:**
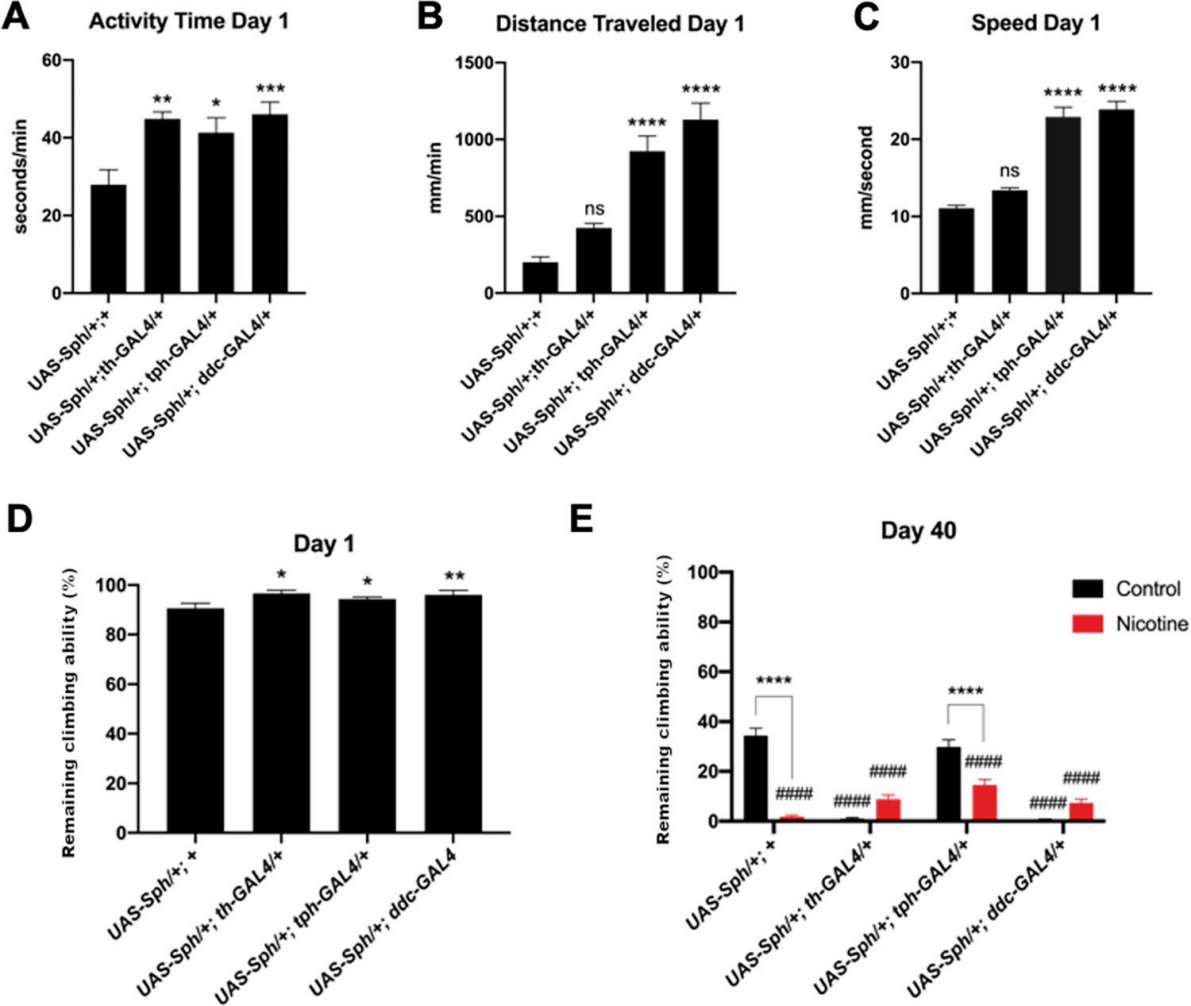
Flies that express Sph-1 in the serotonergic system are hyperactive at day 1 post-eclosion and Sph-1 expression in the dopaminergic system causes age related motility loss. Activity (A), distance traveled (B), and speed (C), three motor parameters, are differentially affected by Sph-1 expression in dopaminergic, serotonergic, or both aminergic populations. D) At day 1 post-eclosion, flies that express Sph-1 show hyperactivity and have an enhanced climbing reflex (negative geotaxis). E) Chronic nicotine treatment (24 μM) for 40 days reduces the climbing ability of control flies (UAS-Sph-1/+; +). Sph-1 expression in the dopaminergic neurons (*UAS-Sph-1/+; UAS-th-GAL4/+* and *UAS-Sph-1/+; UAS-ddc-GAL4/+*) also decreases climbing ability. However, chronic nicotine treatment significantly rescues this phenotype; this nicotine-induced effect is not observed in flies that express Sph-1 only in the serotonergic system (*UAS-Sph-1/+; UAS-tph-GAL4/+*). Significance was tested using two-way ANOVAs followed by a Tukey post-hoc test, n = 40. Asterisks indicate significance between the Sph-1 non-expressing control and the untreated Sph-1 expressing flies, hashtags (#) show significance between nicotine-treated and untreated flies; * = P < 0.05, ** = P < 0.01, *** = P < 0.001, **** = P < 0.0001, ns = not significant, #### = P < 0.001.

Through negative geotaxis experiments, we found that all flies that express Sph-1 in either dopaminergic or serotonergic neurons, or simultaneously in both neuronal populations, exhibit hyperactivity at one day post-eclosion compared to control animals (**Fig 3A–3D**). These data suggest that Sph-1 expression in either of these cell populations affects fly movement in young flies. This increase in activity was also observed using the Buridan tracker (**Fig 3A–3C**).

At 40 days post-eclosion (**Fig 3E**), we observed a natural loss of motility in the control line (*UAS-Sph1/+*) when assessed through negative geotaxis experiments (compare the first black bars in **Fig 3D** and **3E**). This reduced motility is exacerbated after nicotine treatment (**Fig 3E**). In contrast, negative geotaxis experiments in flies expressing Sph-1 in the dopaminergic system (*UAS-Sph1/+; th-GAL4*) showed a greater reduction in motility than the age-matched control line. Motility reduction in these flies was partially rescued by nicotine treatment. On the other hand, flies expressing Sph-1 in serotonergic neurons (*UAS-Sph1/+; tph-GAL4*) did not show any effect on motility compared to the age-matched control line. Interestingly, nicotine treatment reduced the climbing ability of flies expressing Sph-1 in serotonergic neurons, as it was observed in the control strain. Expressing Sph-1 in both dopaminergic and serotonergic neurons (*UAS-Sph1/+; ddc-GAL4*) induced a strong reduction in climbing that was partially rescued by nicotine treatment. This was not significantly different from what we observed in animals expressing Sph-1 only in dopaminergic neurons (**Fig 3E**).

Overall, these results suggest that the loss of climbing ability in Sph-1-expressing animals over time is primarily due to an effect on the dopaminergic system, as motility is only lost when these neurons are compromised (*Sph-1/+; th-GAL4 and Sph-1/+; ddc-GAL4*).

### Sph-1 expression in the serotonergic and the dopaminergic systems induces altered visual perception

Many patients with PD experience visual impairments such as blurry vision, impaired depth perception, and difficulty adjusting to rapid light changes [20]. The Drosophila eye is a well-established model for studying the cellular, physiological, and circuital mechanisms underlying neurodegeneration [21]. In this study, we used electroretinogram studies to evaluate whether the expression of Sph-1 in serotonergic or dopaminergic neurons results in physiological phenotypes in the *Drosophila* visual system that could provide insight into the visual impairments observed in PD patients.

**Fig 4:**
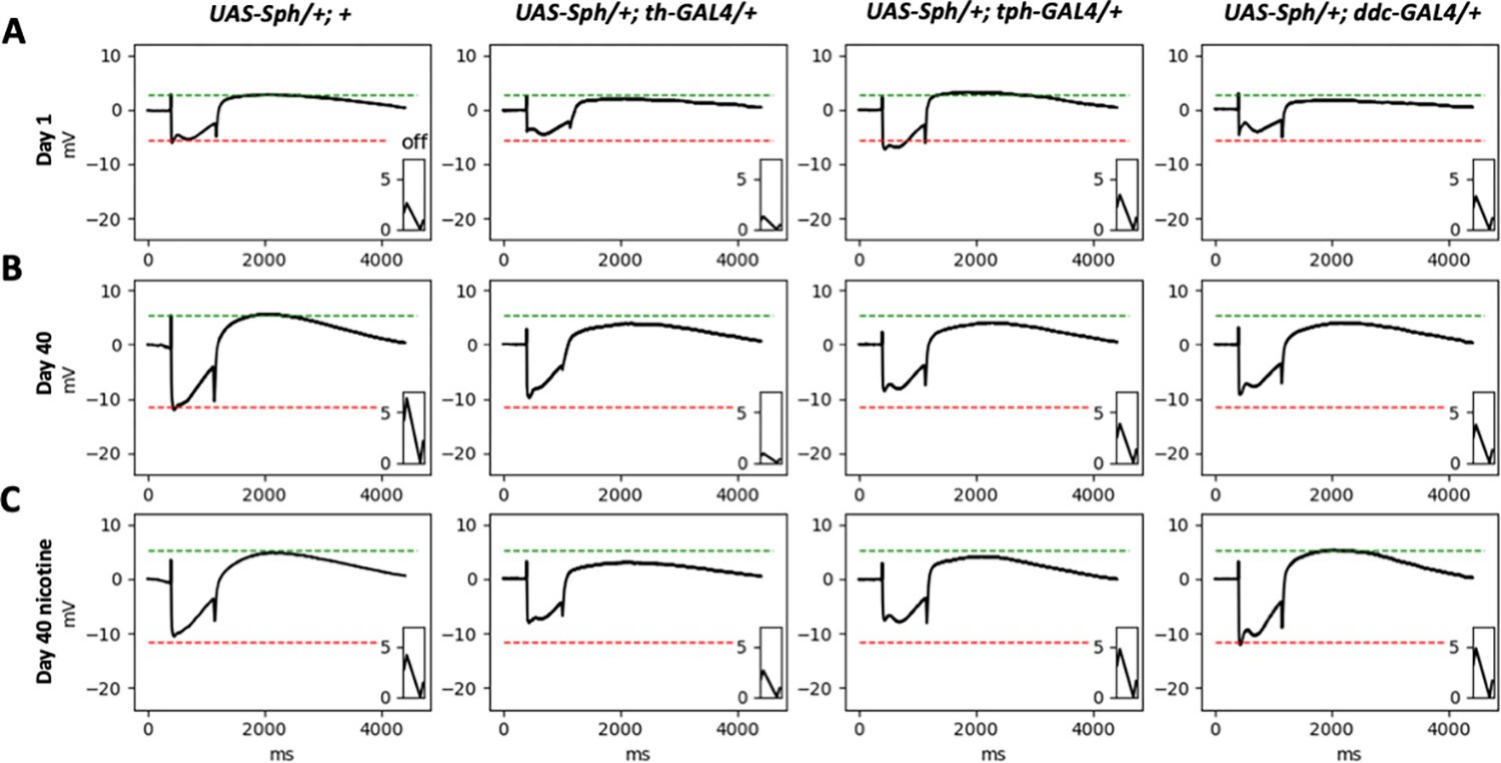
Representative electroretinograms of flies expressing Sph-1 in dopaminergic, serotonergic or in the two aminergic neuronal populations. The electroretinograms were obtained from flies at 1 and 40 days post-eclosion, and represent both nicotine-treated and non-treated animals. The green lines in the figure depict the average magnitude of the "on" transient, while the red lines show the average magnitude of the PRP of the corresponding control line. It is clear from the figure how different genotypes, ages, and treatments can affect the magnitude of these parameters. The parameters that were quantified in this study include the "on" transient (green lines), the "off" transient (inlay), and the PRP (red line). The quantification of these parameters in multiple flies is presented in **Fig 5**.

The parameters measured using an electroretinogram are the "on" and "off" spikes and the amplitude of the photoreceptor response potential (PRP). Electroretinograms were recorded at one and 40 days post-eclosion in control conditions and after nicotine treatment (**Fig 4**).

**Fig 5:**
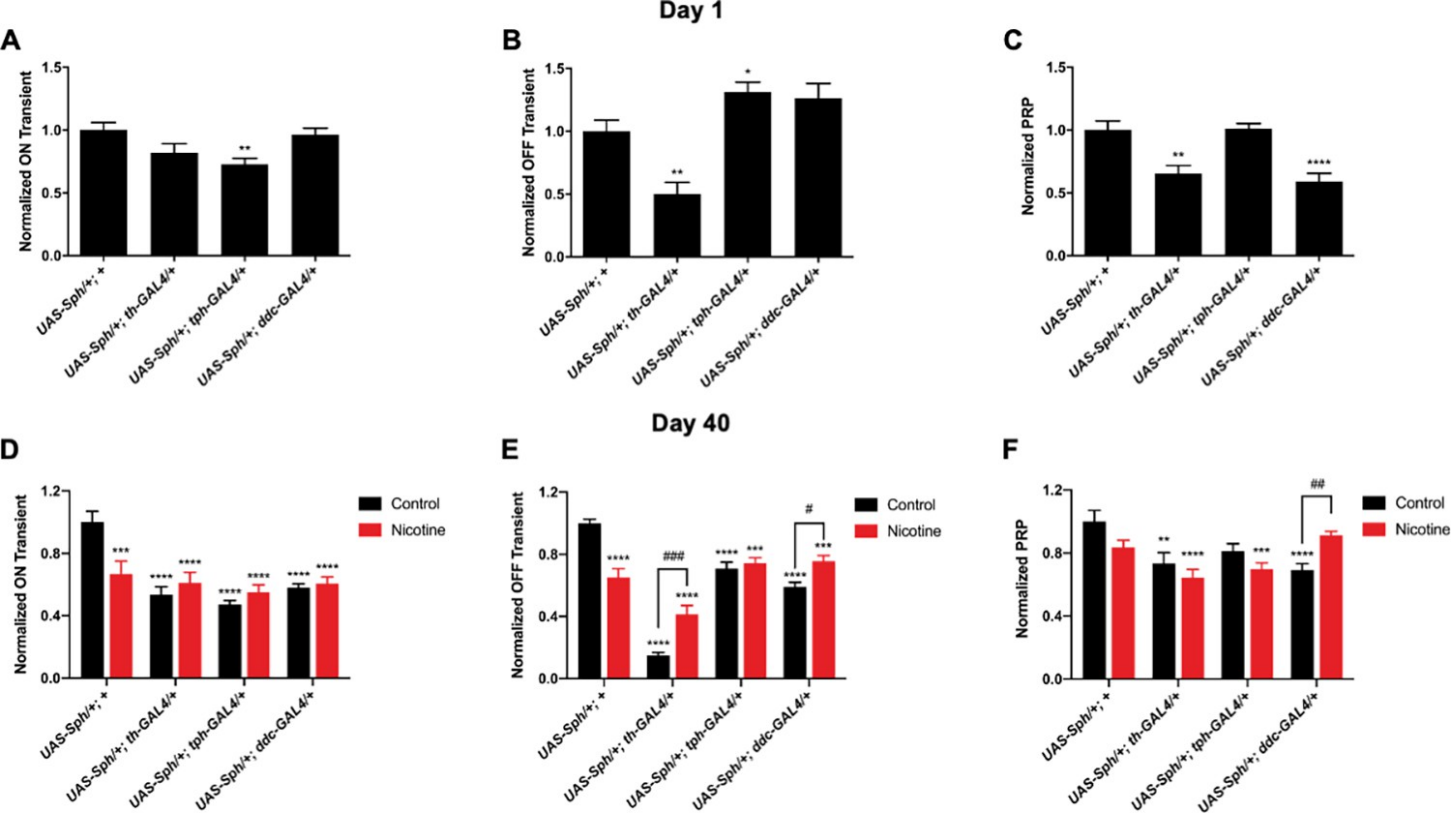
Quantification of the electroretinographic parameters. Panels A-C show the normalized "on," "off," and photoreceptor response potentials (PRP) at one day post-eclosion for the indicated genotypes. All of the flies expressing Sph-1 displayed at least one altered visual parameter compared to the control strain. Panels D-F demonstrate that the expression of Sph-1 in aged flies has a greater effect on visual parameters compared to the control. Nicotine treatment mainly rescues the "off" transient, with a greater effect in the lines expressing Sph-1 in dopaminergic neurons (th and ddc). However, in the *UAS-Sph-1/+; UAS-ddc-GAL4/+* flies, which express Sph-1 in both dopaminergic and serotonergic neurons, the PRP is also rescued. Statistical significance was determined using two-way ANOVAs followed by a Tukey post-hoc test, with n = 20. *p>0.05, **p>0.02, ***p>0.001, ****p>0.0001, ns = not significant.

Our results show that flies that express Sph-1 in either the dopaminergic or the serotonergic systems exhibit electroretinographic alterations at one day post-eclosion. After 40 days post-eclosion, these phenotypes are more evident. Interestingly, nicotine significantly rescued the "off" parameter and had a positive, albeit non-significant, effect on the "on" parameter. Nicotine also significantly rescued the PRP in flies expressing Sph-1 simultaneously in the serotonergic and dopaminergic systems. It is worth noting that nicotine had a detrimental effect on the electroretinographic parameters of the control flies, as has been consistently observed in all previously studied phenotypes (**Fig 5**).

Our data shows that the visual alterations are more evident when Sph-1 is expressed in dopaminergic neurons, either under the control of the TH or the DDC drivers, as the flies age. However, Sph-1 expression in serotonergic neurons has a larger effect on recordings in young flies.

### Sph-1 expression reduces the amount of the rate-limiting enzymes in dopamine and serotonin biosynthesis

**Fig 6:**
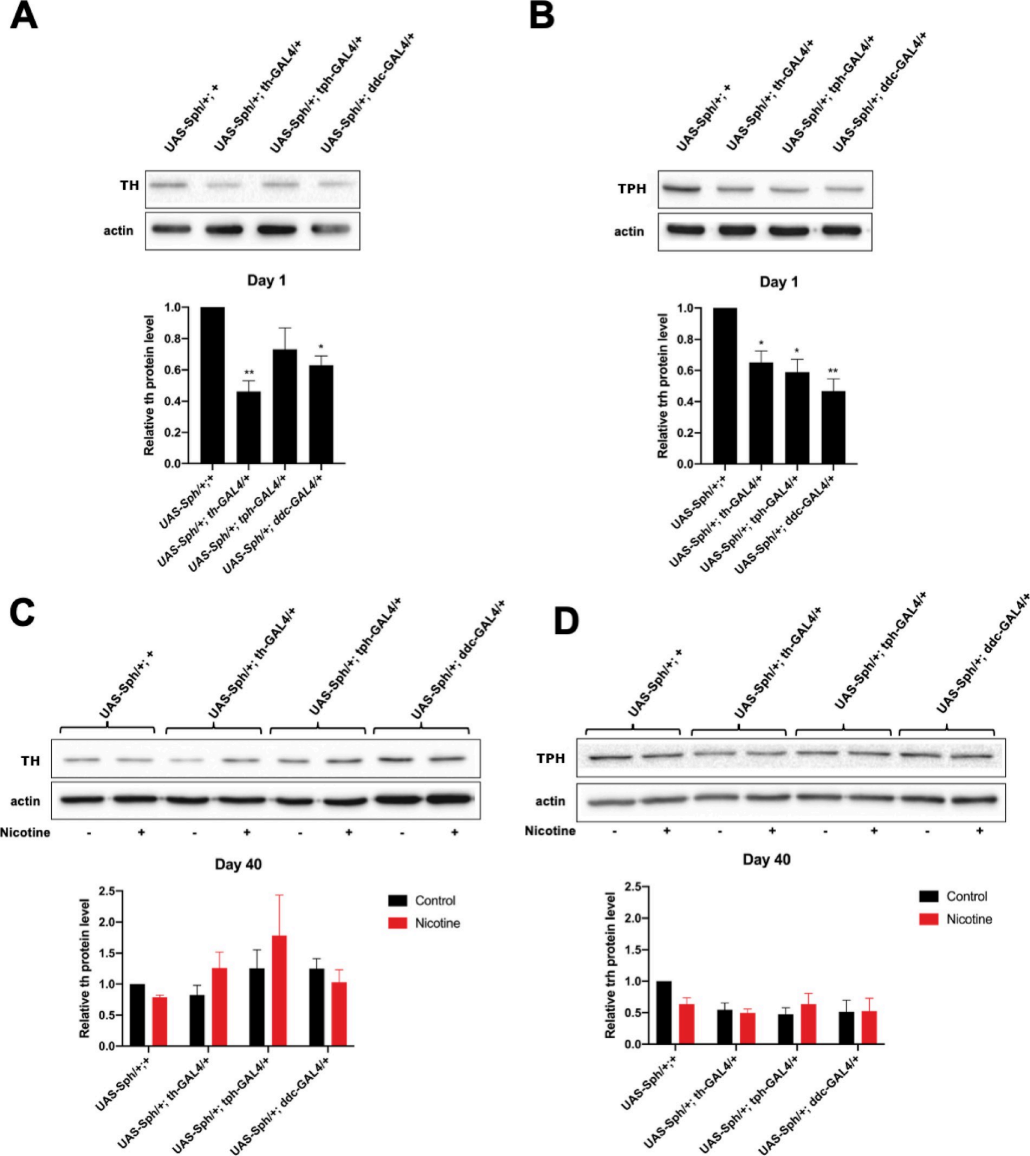
Tyrosine hydroxylase and Tryptophan Hydroxylase levels in nicotine treated and non-treated Sph-1 expressing flies. A) animals expressing Sph-1 in their dopaminergic neurons exhibit a significant reduction in their TH levels at one day post-eclosion. In contrast, flies expressing Sph-1 only in their serotonergic neurons do not show a significant reduction in TH levels. B) Animals expressing Sph-1 in either the serotonergic or dopaminergic system exhibit a significant reduction in TPH expression levels, with a more pronounced effect in flies expressing Sph-1 in both systems (*UAS-Sph-1/+*, *ddc-GAL4/ +*). C) TH expression levels in 40-day-old flies, with the control condition depicted in black and chronic nicotine treatment shown in red. TH levels tend to increase modestly but not significantly in nicotine-treated flies when the serotonergic or dopaminergic system is affected. D) TPH expression levels in 40-day-old flies, with the control condition shown in black and chronic nicotine treatment depicted in red. TPH levels are not affected in nicotine-treated flies. Statistical significance was determined using two-way ANOVAs followed by a Tukey post-hoc test, *p>0.05, **p>0.02, ***p>0.001, ****p>0.0001, ns = not significant, n = 3 independent samples. In all cases experimental animals were treated with 24 μM nicotine, full blots used for quantifications are shown in **S1 and S2 Raw images**.

We previously demonstrated that Sph-1 expression in dopaminergic neurons reduces TH levels [15]. In this study, we measured the protein levels of TPH, the rate-limiting enzyme in serotonin biosynthesis, when Sph-1 is expressed in serotonergic and dopaminergic neurons. At one day post-eclosion, TH levels were reduced in flies expressing Sph-1 in the dopaminergic neuronal population compared to controls. In contrast, no significant change was observed in TH levels in animals expressing Sph-1 only in serotonergic neurons (**Fig 6A**). Interestingly, Sph-1 expression in either aminergic neuronal population resulted in a reduction in TPH levels, and the reduction in TPH expression was more significant when Sph-1 was simultaneously expressed in both aminergic populations (**Fig 6B**). These data suggest that Sph-1 expression in one neuronal type, the serotonergic cell population, leads to deregulation of both aminergic systems. At 40 days post-eclosion, TPH levels were also reduced in the Sph-1 expressing flies; nicotine treatment elicited a modest increase in TPH expression levels, although this change was not statistically significant (**Fig 6C and 6D**).

## Discussion

Serotonin is a well-studied neurotransmitter that has primarily been associated with complex behaviors such as mood control and aggression. Dysregulation of this neurotransmitter has been linked to various mental disorders including autism, schizophrenia, and attention deficit disorder [22]. There is also evidence of important interactions between the dopaminergic and serotonergic systems in both invertebrates and vertebrates [23].

By perturbing the serotonergic system alone (using the *tph-GAL4* driver) or in combination with the dopaminergic system (using the *ddc-GAL4* driver), we were able to distinguish the role of serotonin in the progression of Parkinsonian phenotypes observed in this study. It is known that reduced serotonin in the human brain is involved in several non-motor Parkinsonian phenotypes including fatigue, sleep disorders, depression, and visual impairments [7]. Several studies support the existence of a functional crosstalk between the dopaminergic and serotonergic systems [24–26]. It has also been proposed that long-term perturbations of the serotonergic system may contribute to alterations in dopaminergic circuits underlying the development of motor and non-motor PD symptoms [7,27]. However, the extent to which the dopaminergic and serotonergic neural systems interact functionally in the *Drosophila* brain has not been thoroughly examined. A recent study suggests that this is the case, demonstrating that alteration of the serotonergic system in young animals affects the survival of dopaminergic neurons [28]. Our study advances this issue by showing that serotonergic and dopaminergic systems influence the detection of PD-associated symptoms in flies. Moreover, our data support that nicotine or a molecule with similar pharmacological properties may be a suitable treatment for some of these symptoms, as nicotine is known to increase the amount of available serotonin in the brain [29].

In this study, we used the tryptophan hydroxylase driver (*UAS-Sph1/+; tph-GAL4/+*) to express Sph-1 in serotonergic neurons and the Dopa-decarboxylase driver (*UAS-Sph1/+; ddc-GAL4/+*) to express Sph-1 in both dopaminergic and serotonergic neurons simultaneously [18]. Our results show that lifespan is not affected when Sph-1 is expressed in the serotonergic system, although nicotine induced a small but significant increase in survival in these flies. On the other hand, Sph-1 expression in the dopaminergic system significantly reduced fly survival, and nicotine treatment fully rescued this phenotype. Thus, our data support that a functional dopaminergic system is crucial for normal fly lifespan.

Furthermore, dopamine function is also necessary for the maintenance of motility, as shown in the negative geotaxis experiments. Previous reports indicate that in humans, the serotonergic system is not primarily involved in motility, but rather in more subtle phenotypes such as posture, mood, tremors, and the onset of dementia [7,30]. The non-motor phenotypes evaluated in this study, such as perturbation of the electroretinographic parameters and olfactory impairment, have been proposed as early indicators of PD. Remarkably, these had a deeper penetrance in flies expressing Sph-1 in the serotonergic system than in flies expressing Sph-1 in the dopaminergic system. This allowed us to suggest a differential contribution of these two aminergic neural systems to non-motor PD symptoms.

Electroretinograms were used in our *Drosophila* model to study visual defects associated with PD, something that in other animal models, is not as straightforward as in *Drosophila*. The measurement of the effect of Sph-1 expression on the ON and OFF parameters provides information about postsynaptic potentials, while the PRP reports the effect of different experimental conditions on photoreceptor cells [31,32]. Our data show that Sph-1 independently expressed in dopaminergic or serotonergic neurons affects the postsynaptic component of the electroretinogram from day 1 and this perturbation increases with aging. The simultaneous expression of Sph-1 in dopaminergic and serotonergic neurons strongly affects PRP, probably because Sph-1 is damaging the serotonergic and dopaminergic neurons of the retina and the lamina that modulate photoreceptors; importantly, this phenotype is completely reverted with nicotine treatment at day 40. It has been reported that intrinsic neurons of the lamina express serotonergic receptors and serotonin transporters and that both, receptor and transporters, are essential for proper eye development and maturation [33,34]. Our data also show that Sph-1 expression only in the serotonergic system has a profound effect on vision, possibly due to a lack of serotonin modulation of the neurons in the lamina. The fact that nicotine rescues the ON, OFF and PRP parameters in flies that express Sph-1 either on the serotonergic or the dopaminergic or in both neural systems simultaneously suggests a crosstalk between these two systems that converge into a cholinergic signal. It has been reported that there are cholinergic neurons in the lamina, further supporting the hypothesis that there is a convergence of the serotonergic and dopaminergic signals in this tissue [35]. Our ERG experiments show that the photoreceptor signal is perturbed in Sph-1 expressing flies, and their postsynaptic signal to the lamina is also perturbed or disrupted, suggesting that nicotine bypasses the serotonergic and dopaminergic signals in this tissue and rescues the postsynaptic phenotype. Although there are no reports of acetylcholine participating in the ERG response, our data suggest that in wild type organisms there is a cholinergic component, as nicotine (a canonical cholinergic agonist) is sufficient to rescue the ERG parameters affected by Sph-1 expression.

Overall, our data support the notion that olfactory loss and perturbations of the visual system in PD have a strong serotonergic component, in concordance with reports that in PD patients there is neuronal death of the serotonergic neurons that project to the striatum and to some regions of the *sustancia nigra pars reticulata* from the *raphe nuclei*. This is further supported by the fact that Lewy bodies have been identified in the area of the *raphe nuclei*, which is known to be highly enriched in serotonergic neurons [30,36]. These reports suggest that a reduction in serotonin in the brain may contribute to a reduction in available dopamine levels, significantly contributing to the onset and severity of PD symptoms. Nicotine probably rescues olfaction by augmenting the amount of available serotonin or by supplying cholinergic stimulation. This is relevant because in humans cholinergic signaling has a central role for odor perception [37] and it has been demonstrated that in *Drosophila* [15,38] and mouse [39] PD models nicotine rescues olfaction and in rats it promotes olfactory bulb activity [40]. Interestingly, we found an inverse relationship between the expression levels of the rate-limiting enzymes for the synthesis of serotonin and dopamine, and the expression of Sph-1 in these neuronal populations. This further supports the idea that there is crosstalk between both circuits in young and aged flies. The western blot data we present suggest that the dopaminergic circuit does interact with the serotonergic one. As dopamine is an excitatory neurotransmitter, it is likely that dopaminergic stimulation of the serotonergic circuitry increases its activity and TPH synthesis, which is necessary for the production of serotonin. Conversely, a lack of dopaminergic stimulation (such as due to dopaminergic circuitry depression or dopaminergic neuron loss) would likely result in a decrease in serotonergic activity and TPH synthesis. It has been reported that, over development, serotonin has a fundamental role in the control of normal dopaminergic innervation patterns, and that abnormal serotonergic function is correlated with the development of schizophrenia, depression, and PD. This has also been demonstrated in *Drosophila*, where imbalanced dopamine/serotonin signaling leads to extensive neuroanatomical adaptations in mutants that lack neural dopamine. Similarly, in rodent models of PD, a lack of dopamine leads to increased serotonin levels and arborizations in specific brain regions. On the other hand, increased dopamine levels through L-DOPA feeding result in reduced connectivity of serotonin neurons to their target neurons in the mushroom body (MB). These alterations in serotonin neuron plasticity suggest that the loss of DA signaling is not the sole cause of behavioral disorders observed in Drosophila models of PD, but rather a combination of this loss with changes in the serotonin circuitry [41]. These changes in circuitry may affect other behaviors, such as feeding, grooming, or aggression, and may differentially affect survival without causing other phenotypes, like motor defects.

On the other hand, it is also known that dopamine depletion during development results in defective serotonergic innervation, supporting the hypothesis that during human development there is crosstalk between the serotonergic and dopaminergic circuits. This also provides support for the possibility that when this crosstalk is affected, it could lead to pathogenic processes such as PD [42].

There are several possible explanations for the fact that nicotine is only beneficial in Parkinsonian genotypes, this effect has been observed in our model and in other *Drosophila* PD models such as Parkin mutant flies [38]. One possibility is that nicotine acts on the nicotinic acetylcholine receptors (nAChRs) present in both wild type and mutant *Drosophila*. These receptors are widely distributed in the brain and nervous system and play a role in a variety of functions, including learning, memory, and movement. Activation of nAChRs can lead to the release of neurotransmitters such as dopamine and serotonin, which can affect behaviors such as locomotion. It is possible that in wild type *Drosophila*, chronic exposure to nicotine may result in overstimulation of nAChRs and an excess release of neurotransmitters, leading to excitotoxicity or other negative effects that would affect lifespan and motor abilities. On the other hand, in mutant *Drosophila* expressing mutations associated with Parkinson’s disease, the dopaminergic system may be impaired, leading to a deficiency in dopamine signaling. In this case, nicotine-induced activation of nAChRs may compensate for the dopamine deficiency and improve lifespan and motor abilities.

Another possibility is that nicotine may have antioxidant effects that protect against oxidative stress, which has been linked to aging and neurodegenerative disorders such as Parkinson’s disease. It is possible that the observed effects of nicotine on lifespan and motor abilities in *Drosophila* may be mediated, at least in part, by its ability to reduce oxidative stress.

It is also possible that the observed effects of nicotine may be due to other mechanisms, such as modulation of gene expression or signaling pathways involved in aging and neurodegeneration. Further research would be needed to fully understand the molecular and cellular mechanisms underlying the observed effects of nicotine on *Drosophila*.

Finally, it is important to stress the beneficial effects of nicotine in the parkinsonian genotypes: our result strongly suggest that nicotine or some other cholinergic agonists with better pharmacological properties may be used as palliatives for several parkinsonian symptoms.

## Materials and methods

### Animals

All experiments were performed with male flies. Flies were obtained from the Bloomington *Drosophila* Stock center, and experimental flies were generated by crossing homozygous males that have the following drivers (*+; th-GAL4/th-GAL4*, Stock No. 8848, +; *tph-GAL4/tph-GAL4*, Stock No. 38389 or +; *ddc-GAL4/ddc-GAL4*, Stock No. 7010) with the *UAS-Sph* strain that we previously generated and characterized, which expresses the canonical 919aa Sph-1 isoform [14]. UAS-*Sph/+; +/+* flies that do not express Sph-1 were used as controls, and were obtained by crossing male homozygous *UAS-Sph* flies with w^1118^ females. All experiments were performed at a constant temperature and light conditions (25°C and a 12h/12h light/dark cycle).

### Nicotine treatment

All flies were reared on standard cornmeal yeast food. When necessary, food was supplemented with nicotine base (Sigma) at a final concentration of 24 μM, which was previously determined to be the most effective concentration for flies with a parkinsonian (Sph-1 expressing) genotype [15]. Treated and untreated flies were kept in their respective conditions throughout their lifetimes and were transferred to a fresh vial every third day.

### Survival

One hundred male flies were selected at day 1 post-eclosion and divided into groups of 10 animals, placed in vials containing either regular (control) or nicotine-supplemented (experimental) food. The number of dead flies in each group was counted every 4 days until there were no surviving individuals. Survival was analyzed using the Kaplan-Meier estimator and evaluated using the Mantel-Cox test.

### Behavioral assays

All behavioral experiments (negative geotaxis experiments and olfactory performance assays) were double blinded, meaning that the researcher that performed the behavioral experiment was unaware of the specific genotype and treatment being tested. One researcher randomly assigned code numbers to each group of flies (for negative geotaxis) or for each individual fly (for olfactory performance), while another researcher performed the behavioral test using the code numbers to identify each group or fly. The resulting data from the behavioral test was analyzed and then ascribed to the corresponding genotypes and treatments after the completion of all experiments. This process minimizes any biases that may affect the results of the study.

### Negative geotaxis (motility)

Motility was quantified using the negative geotaxis paradigm [43]. Briefly, thirty flies per group were deposited in groups of ten animals in empty *Drosophila* vials. Flies were dropped to the bottom of the vial with a gentle tap and the ones that were able to climb 5 cm in 10 seconds or less were counted, the assay was repeated 10 times per group with a 1 min resting period in between. Negative geotaxis experiments were performed with flies of the indicated genotypes and treatments at days 1 and 40 post-eclosion.

### Olfactory assays

Olfactory performance was evaluated as previously described [44]. Briefly, single flies were placed in a circular arena (disposable Petri dishes 39 mm in diameter and 2 mm in height). For each experiment a new Petri dish was used and then disposed. Flies were exposed to a repulsive olfactory stimulus for 3 min (100 μl of a 10% solution of benzaldehyde in a small cotton ball) on one side of the arena, while the opposite side contained no stimulus (another cotton ball soaked in 100 μl of water). The side where the benzaldehyde stimulus was located was chosen randomly for each individual/experiment to avoid any arena positional bias. The open software Buritrack was used to quantify the olfactory performance index (P.I). The P.I. is a measure of a fly’s innate avoidance of benzaldehide, which is determined by dividing the heat map of the time spent by the fly over the area of the arena produced by Buritrack into two halves and using the formula P.I. *=* AUCH_2_O-AUCBz/ AUCH_2_O+AUCBz, where AUCH2O = area under curve of time spent in the water half, and AUCBz = area under curve of time spent in the Bz half. Twenty flies of each genotype were evaluated. Animals at day 1 post-eclosion were studied because it has already been reported that only young flies expressing Sph-1 exhibit alterations in their response to the olfactory stimulus [15].

### Electroretinograms

For electroretinogram (ERG) studies, flies were embedded in an electrocardiogram gel (ElectroGel 0992E95 SSA) with the head exposed. The reference electrode was placed directly on the gel, and the recording electrode was filled with a 30mM tricholine citrate solution (Sigma) and placed in contact with the fly’s eye to obtain the recording. The light stimulus lasted 1 s. The generated signals were amplified using a DC differential amplifier (A-M Systems, Carlsborg, WA), displayed, and recorded on a digital oscilloscope using the DBWAVE software [45]. The data presented in this work correspond to electrophysiological recordings obtained from at least 20 flies of each genotype, treatment, and age.

### Western blot analysis

Total fly head extracts were prepared in 250 mM sucrose, 5 mM Tris pH 7.5, 25 mM KCl, 5 mM MgCl2, 5 mM EDTA, in 1X Complete Protease Inhibitor Cocktail (Roche), and 100 mM PMSF (Sigma) for semi-quantitative analysis. Fifty μg of total protein were loaded onto 12% SDS polyacrylamide gels (Biorad Mini protean gel electrophoresis system) and transferred to nitrocellulose membranes for 3 hrs at 250 mA in Tris 25 mM, Glycine 190 mM, and 20% methanol. Tyrosine hydroxylase (TH) was detected as follows: membranes were blocked overnight at 4°C in PBST (0.1% Tween 20) supplemented with 10% powdered milk. Blocked membranes were then incubated overnight at 4°C with anti-TH (Inmunostar 1:1000) in PBST + 5% milk. Membranes were then washed 3 times with PBST. Tryptophan hydroxylase (TPH) was detected as follows: membranes were blocked overnight at 4°C in PBST (0.1% Tween 20) supplemented with 5% BSA (Roche). Blocked membranes were then incubated overnight at 4°C with anti-TPH (Micropore 1:1000) in PBST + 5% BSA. Membranes were then washed 6 times with PBST. Then, membranes were incubated 1h at room temperature with HPRT goat-anti-mouse (SeraCare 1:3000) or HPRT anti sheep (Zymed 1:3000) in PBST supplemented with 5% powdered milk. Membranes were then washed 6 times with PBST and bands were detected using the kit SuperSignal West pico Chemiluminiscence Substrate (ThermoFisher) following manufacturer’s instructions. Blot signals were digitized and quantified using ImageJ. Anti-actin (DHB, 1:3000) was used as internal loading control. Images of the blots used for densitometry assays can be found in **S1 and S2 Raw images**. Western blots used to quantify TH and TPH in the different genotypes ages and treatments.

### Experimental design and statistical analysis

Differences between groups were assessed using ANOVA and Tuckey’s post-hoc test. Survival (life expectancy) was analyzed using the Mantel-Cox log-rank test, and survival of aged flies (older than 80 days) was analyzed using Fisher’s exact test. Average data is plotted with the corresponding standard error of the mean. Significance was defined as p < 0.05. All data were analyzed using GraphPad Prism 8 software. Sample size was based on previous similar studies and is indicated in the corresponding figure footnotes. All experiments were performed with male flies. P-values are indicated as follows: * P < 0.05, ** P < 0.01, *** P < 0.001, **** P < 0.0001; # P < 0.05, ## P < 0.01, #### P < 0.0001.

### Ethical approval

No human or vertebrates were used in this work. The project was approved by the Bioethical Committee of the Instituto de Biotecnología and performed accordingly to the ethical guidelines of our institution.

## Supporting information

S1 Raw images (TIF)

S2 Raw images (TIF)
