# Supplementary figures and images for: Parkinsonian phenotypes induced by Synphilin-1 expression are differentially contributed by serotonergic and dopaminergic circuits and suppressed by nicotine treatment
Source: PLoS One. 2023 Mar 1;18(3):e0282348. doi: 10.1371/journal.pone.0282348 (PMC9977059; doi:10.1371/journal.pone.0282348)

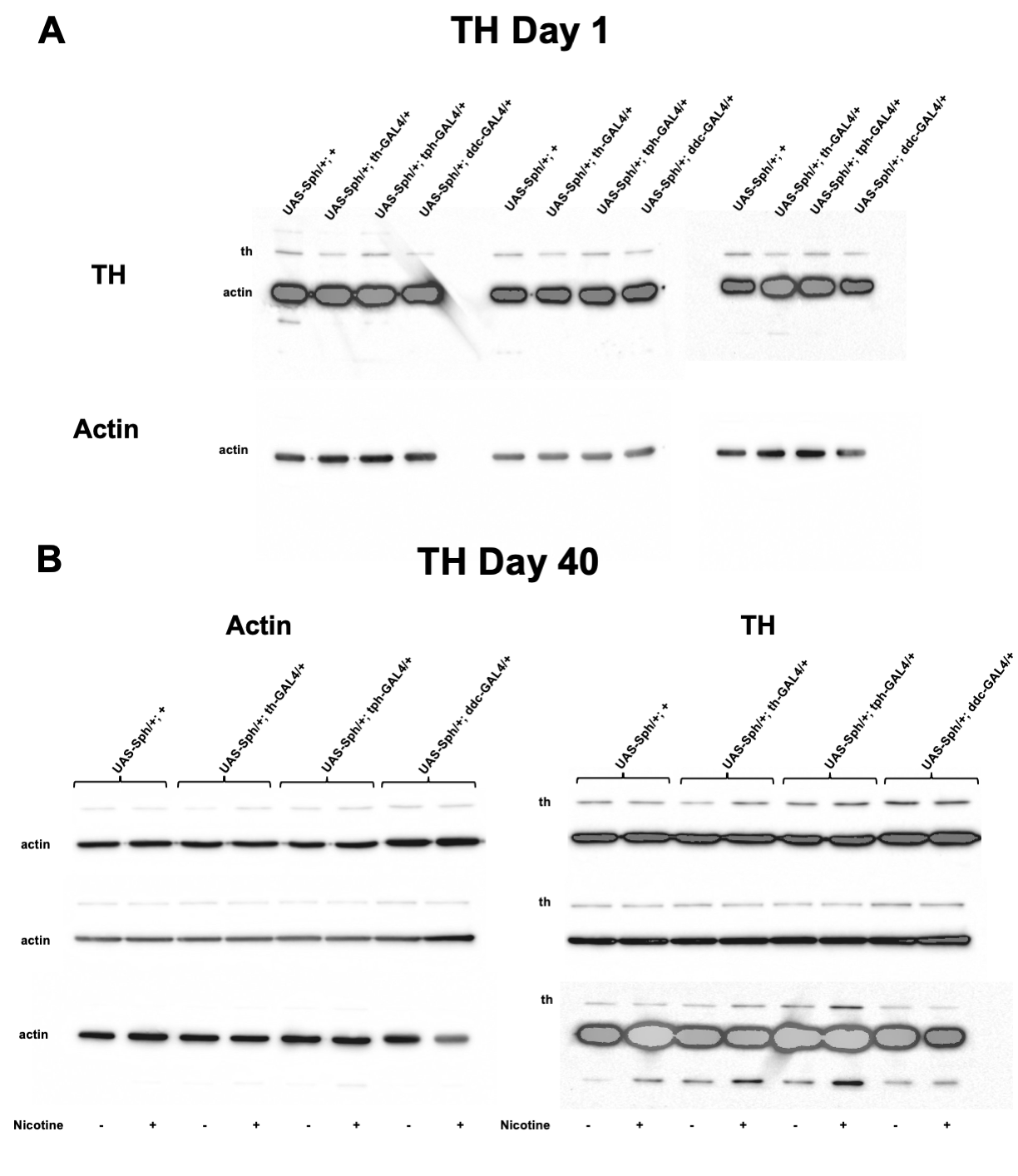

Supplement: S1 Raw images — (TIF) [file pone.0282348.s001.tif]

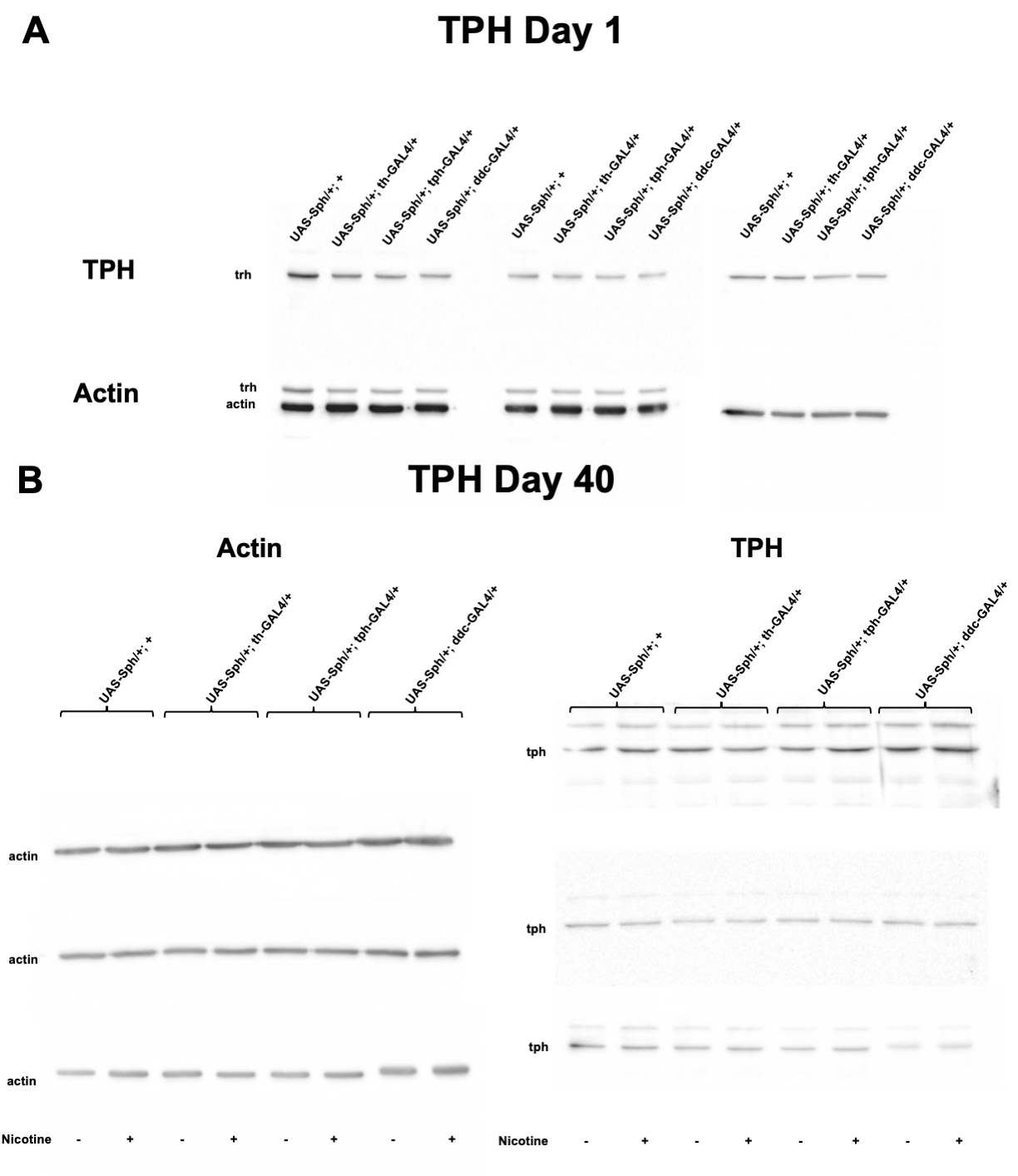

Supplement: S2 Raw images — (TIF) [file pone.0282348.s002.tif]
